# Regulating role of fetal thyroid hormones on placental mitochondrial DNA methylation: epidemiological evidence from the ENVIR*ON*AGE birth cohort study

**DOI:** 10.1186/s13148-017-0366-y

**Published:** 2017-06-21

**Authors:** Bram G. Janssen, Hyang-Min Byun, Harry A. Roels, Wilfried Gyselaers, Joris Penders, Andrea A. Baccarelli, Tim S. Nawrot

**Affiliations:** 1 Centre for Environmental Sciences, Hasselt University, Agoralaan Building D, 3590 Diepenbeek, Belgium; 2 Human Nutrition Research Centre, Institute of Cellular Medicine, Newcastle University, Newcastle upon Tyne, NE4 5PL UK; 3 Louvain Centre for Toxicology and Applied Pharmacology (LTAP), Université catholique de Louvain, Brussels, Belgium; 4 Department of Obstetrics, East-Limburg Hospital, Genk, 3600 Belgium; 5 Biomedical Research Institute, Hasselt University, Diepenbeek, 3590 Belgium; 6 Laboratory of Clinical Biology, East-Limburg Hospital, Genk, 3600 Belgium; 7 Department of Environmental Health Sciences, Mailman School of Public Health, Columbia University, New York, NY 10032 USA; 8 Department of Public Health & Primary Care, Occupational & Environmental Medicine, Leuven University, Leuven, Belgium

**Keywords:** DNA methylation, Epigenetics, Mitochondria, Mitochondrial DNA content, Placenta, Fetal thyroid hormones

## Abstract

**Background:**

Fetal development largely depends on thyroid hormone availability and proper placental function with an important role played by placental mitochondria. The biological mechanisms by which thyroid hormones exert their effects on mitochondrial function are not well understood. We investigated the role of fetal thyroid hormones on placental mitochondrial DNA (mtDNA) content and mtDNA methylation. We collected placental tissue and cord blood from 305 mother–child pairs that were enrolled between February 2010 and June 2014 in the ENVIR*ON*AGE (ENVIRonmental influence *ON* early AGEing) birth cohort (province of Limburg, Belgium). Placental mtDNA content was determined by qPCR and placental mtDNA methylation by bisulfite-pyrosequencing in two regions, i.e., the *D*-*loop* control region and 12S ribosomal RNA (*MT-RNR1*). The levels of free thyroid hormones (FT_3_, FT_4_) and thyroid-stimulating hormone (TSH) were measured in cord blood.

**Results:**

Cord blood FT_3_ and FT_4_ were inversely associated with placental mtDNA methylation at the *MT-RNR1* (*p* ≤ 0.01) and *D-loop* (*p* ≤ 0.05) regions, whereas a positive association was observed for both hormones with placental mtDNA content (*p* ≤ 0.04). Assuming causality, we estimated that *MT-RNR1* and *D-loop* methylation mediated, respectively, 77% [indirect effect +14.61% (95% CI 2.64 to 27.98%, *p* = 0.01)] and 47% [indirect effect +8.60% (95% CI 1.23 to 16.50%, *p* = 0.02] of the positive association between FT_3_ and placental mtDNA content. Mediation models with FT_4_ gave similar results but the estimated effect proportions were smaller compared with those of FT_3_ (54% and 24%, respectively).

**Conclusions:**

We showed that epigenetic modification at specific loci of the mitochondrial genome could intervene with the thyroid-dependent regulation of mitochondrial DNA copy numbers.

**Electronic supplementary material:**

The online version of this article (doi:10.1186/s13148-017-0366-y) contains supplementary material, which is available to authorized users.

## Background

Thyroid hormones are known to have profound effects on mitochondrial energetics and biogenesis [1, 2]. Under the control of thyroid-stimulating hormone (TSH), the thyroid gland produces thyroxine (T_4_), the major form of thyroid hormone, and triiodothyronine (T_3_), the active form. On account of its biological activity, unbound or free T_3_ (FT_3_) regulates mitochondriogenesis most likely in two different cell compartments: (i) binding of FT_3_ to thyroid receptors (TRs) that consecutively bind to response elements in the nucleus, activating the expression of mitochondria-related genes such as peroxisome proliferator-activated receptor γ-coactivator1α (*PPARGC1A*) or (ii) direct binding to specific TRs in the mitochondrial matrix stimulating transcription of the mitochondrial genome [3].

The placenta is a relevant tissue to investigate the interplay between thyroid hormones and mitochondria. This metabolically active organ contains a high number of mitochondria and regulates nutrient, oxygen, and hormonal transfer, allowing for fetal growth. It is also well established that thyroid hormones are critical for placental [4] and fetal development [5]. FT_3_ stimulates the production of factors that control trophoblast growth and development [6]. The placenta expresses two types of iodothyronine deiodinases (D2 and D3) that are capable of metabolizing FT_4_ to FT_3_ [7], and thus, plays an important role in thyroid hormone homeostasis.

Although some studies are questioning the presence of DNA methylation in the mitochondrial genome [8, 9], in the last few years, mtDNA methylation has emerged as a next-generation biomarker that might be a diagnostic tool for several diseases such as cardiovascular disease [10]. Here, we examined two specific mtDNA loci, i.e., the displacement loop (*D-loop*) and *MT-RNR1*. DNA methylation levels in the *D-loop* control region are suggested to play an important role in modulating either the replication or transcription of mtDNA since nearly the entire mitochondrial genome transcribes from this region [11]. Our other studied locus *MT-RNR1* encodes for the 12S rRNA protein which is critical for normal function and integrity of the mitochondrial ribosome [12]. In this mother–newborn study, we aimed to elucidate possible mechanisms involved in the regulation of mitochondrial biogenesis by investigating the association between fetal FT_3_, FT_4_, and TSH with mtDNA content and mtDNA methylation in placental tissue. We hypothesized that methylation at specific loci of the mitochondrial genome could interfere with thyroid hormone-dependent regulation of mitochondrial biogenesis.

## Results

### Mother–newborn characteristics and demographics

The study included 305 mother–child pairs (mean maternal age, 29.1 yr; range, 18–42 years). Demographic and prenatal lifestyle factors are reported in Table 1. Briefly, mean (10th–90th percentile) pre-pregnancy body mass index (BMI) of the participating mothers was 24.1 (19.7–29.7) kg/m^2^. Fifty-two mothers (17.1%) reported to have smoked during pregnancy and smoked on average 7.8 cigarettes per day. Most women (64.9%, *n* = 198) never smoked cigarettes. The majority (>50%) of the mothers were highly educated. Nearly half of the newborn population were boys (*n* = 151; 49.5%). The overall mean gestational age was 39.2 weeks (10th–90th percentile 18–41) and included a vast majority of primiparous (51.8%, *n* = 158) or secundiparous (36.7%, *n* = 112) newborns.

**Table 1:** Characteristics of mother–child pairs (*n* = 305)

Characteristics	Mean (10th–90th percentile) or frequency (%)	
Mother		
Age, years	29.1	(23–36)
Pre-pregnancy BMI, kg/m^2^	24.1	(19.7–29.7)
Net weight gain, kg	14.7	(7.5–22.0)
Maternal education^a^		
Low	37	(12.1%)
Middle	107	(35.1%)
High	161	(52.8%)
Self-reported smoking habit		
Never smoker	198	(64.9%)
Cessation before pregnancy	55	(18.0%)
Smoker during pregnancy	52	(17.1%)
Parity		
1	158	(51.8%)
2	112	(36.7%)
≥3	35	(11.5%)
Newborn		
Sex		
Male	151	(49.5%)
Ethnicity^b^		
European-Caucasian	269	(88.2%)
Gestational age, weeks	39.2	(38–41)
Season at delivery		
Winter (Dec–Mar)	80	(26.2%)
Spring (Mar–Jun)	89	(29.2%)
Summer (Jun–Sep)	60	(19.7%)
Autumn (Sep–Dec)	76	(24.9%)
Apgar score after 5 min		
7 or 8	20	(6.6%)
9	90	(29.5%)
10	195	(63.9%)
Cord plasma insulin, pmol/L^*^	46.4	(15.3–85.4)
Birth weight, g	3421	(2880–3985)

^a^Mother’s education: low (no high school diploma), middle (high school diploma), high (college or university diploma)

^b^Based on the native country of the newborn’s grandparents. European-Caucasian when two or more grandparents were European, or non-European when at least three grandparents were of non-European origin ^*^Geometric mean

### Levels of fetal thyroid hormones, placental mtDNA methylation, and mtDNA content

**Fig. 1:**
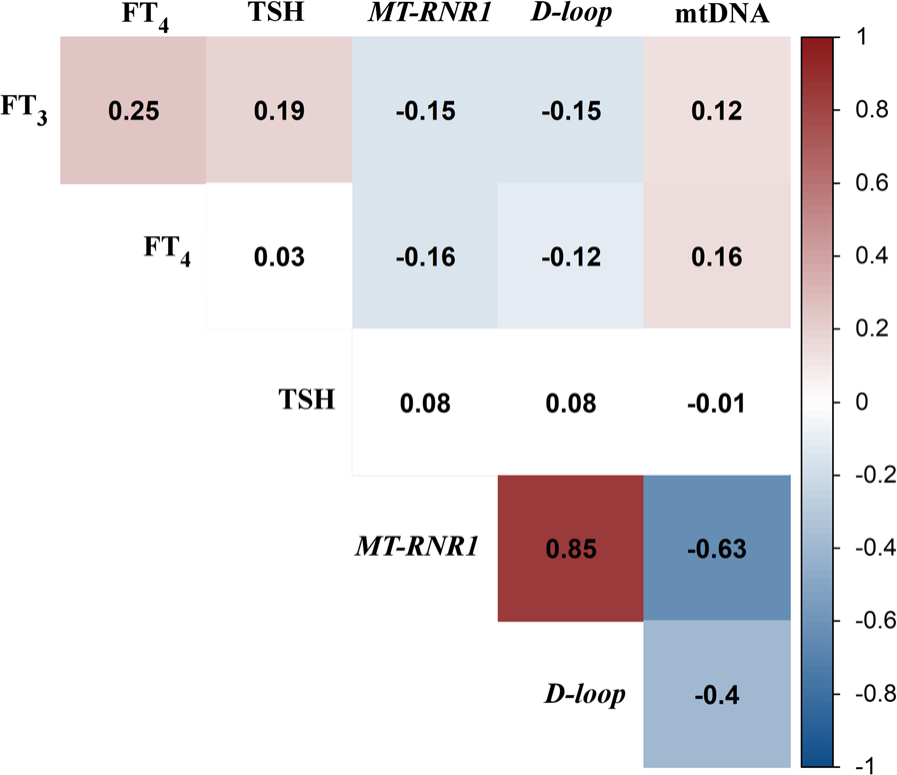
Unadjusted correlation matrix between thyroid hormones (FT_3_, FT_4_, TSH), mtDNA methylation (*MT-RNR1*, *D-loop*), and mtDNA content. *Numbers* represent Pearson correlation coefficients and only the *colored boxes* are significant correlations at *p-*level < 0.05 (*red*: positive significant correlation; *blue*: negative significant correlation; *blank*: not-significant)

The geometric means (10th–90th percentile) of thyroid hormone levels in cord blood were 2.63 (2.15–3.22) pmol/L for FT_3_, 15.66 (13.26–18.53) pmol/L for FT_4_, and 11.65 (5.16–20.59) mU/L for TSH (Table 2). A positive correlation was observed between the FT_3_ and FT_4_ values (*r* = 0.25; *p* < 0.0001) and between the FT_3_ and TSH values (*r* = 0.19; *p* = 0.0008) (Fig. 1).

**Table 2:** Cord blood thyroid hormone levels and placental mtDNA content and mtDNA methylation

	Mean	SD	10th percentile	90th percentile
Thyroid hormones^a^				
FT_3_, pmol/L	2.63	0.47	2.15	3.22
FT_4_, pmol/L	15.66	2.08	13.26	18.53
TSH, mU/L	11.65	7.60	5.16	20.59
mtDNA methylation^b^				
*MT-RNR1*, %	9.51	4.19	4.46	14.67
*D-loop*, %	3.61	1.31	1.94	5.24
mtDNA content (unitless)^b,c^	1.11	0.96	0.49	2.49

Values are presented as geometric means with 10th-90th percentiles, except for mtDNA methylation for which the arithmetic mean is given

^a^Laboratory reference values for adults range from 4.0 to 6.8 pmol/L for FT_3_, from 12.0 to 21.9 pmol/L for FT_4_, and from 0.3 to 4.2 mIU/L for TSH

^b^Measured in placental tissue

^c^Determined as the ratio of two mitochondrial genes gene copy numbers (*MTF3212/R3319* and *MT-ND1*) to two single-copy nuclear control genes (*RPLP0* and *ACTB*)

Table 2 also shows mtDNA methylation and mtDNA content values measured in placental tissue. Placental mtDNA methylation averaged (10th–90th percentile) 9.51% (4.46–14.67) for *MT-RNR1* and 3.61% (1.94–5.24) for *D-loop*. The methylation levels of *MT-RNR1* and *D-loop* were strongly correlated (*r* = 0.85, *p* < 0.0001) (Fig. 1 and Additional file 1: Figure S1). The geometric mean of placental mtDNA content was 1.11 (0.49–2.49) (unitless). In this subset, and as previously published [13], we report a strong inverse correlation between placental mtDNA methylation and mtDNA content (*r* = −0.63, *p* < 0.0001 for *MT-RNR1* and *r* = −0.40, *p* < 0.0001 for *D-loop*) (Fig. 1).

### Association of fetal thyroid hormones with placental mtDNA methylation and mtDNA content

Both fetal FT_3_ and FT_4_ were inversely correlated with mtDNA methylation at the *MT-RNR1* and *D-loop* region, whereas a positive correlation was observed for both hormones with mtDNA content in placental tissue (see Fig. 1 for correlations). Fetal TSH levels did not correlate with either placental mtDNA methylation or mtDNA content.

The associations remained significant after adjustment for maternal age, pre-pregnancy BMI, gestational age, newborn’s sex, smoking status, parity, maternal education, ethnicity, and cord plasma insulin level. A 10th–90th percentile increment (53%) in cord blood FT_3_ (log_10_ values) was associated with a lowering in absolute methylation of −1.50% (95% CI −2.70 to −0.30%, *p* = 0.01) for *MT-RNR1* and −0.46% (95% CI −0.83 to −0.09%, *p* = 0.02) for *D-loop*. A similar association, but with smaller estimates, was observed for FT_4_ (Table 3). On the other hand, a 10th–90th percentile increment in cord blood FT_3_ (53%) or FT_4_ (13%) was associated with a higher placental mtDNA content (relative change) of +20.35% (95% CI 0.47 to 44.17%, *p* = 0.04) for FT_3_ and +11.04% (95% CI 4.00 to 18.54%, *p* = 0.002) for FT_4_.

**Table 3:** Associations of placental mtDNA methylation and content with cord blood thyroid hormones

	FT_3_, pmol/L		FT_4_, pmol/L		TSH, mU/L	
Variable	β	(95% CI)	β	(95% CI)	β	(95% CI)
mtDNA methylation^a^						
*MT-RNR1*	-1.50	(-2.70 to -0.30)^*^	-0.60	(-1.04 to -0.16)^*^	0.38	(-0.21 to 0.97)
*D-loop*	-0.46	(-0.83 to -0.09)^*^	-0.14	(-0.28 to -0.002)^*^	0.11	(-0.07 to 0.29)
mtDNA content^b^	20.35	(0.47 to 44.17)^*^	11.04	(4.00 to 18.54)^**^	-5.34	(-13.35 to 3.40)

^a^β represents an absolute change in placental mtDNA methylation percentage (%) for a 10th-90th percentile increment of cord blood thyroid hormone

^b^β represents a relative change (%) in placental mtDNA content for a 10th-90th percentile increment of cord blood thyroid hormone

All models are adjusted for maternal age, pre-pregnancy BMI, gestational age, newborn’s sex, smoking status, parity, maternal education, ethnicity, and cord plasma insulin level

^*^ *p*-value < 0.05, ^**^ *p-*value *<* 0.005

### Mediation analysis

**Fig. 2:**
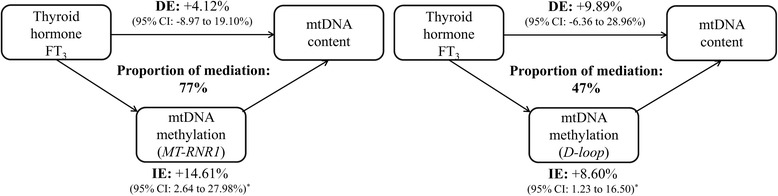
Estimated proportion of effects of FT_3_ exposure on mtDNA content mediated by mtDNA methylation. The figure displays placental mtDNA methylation as mediator (*left panel*: *MT-RNR1*; *right panel*: *D-loop*), the estimates of indirect effect (IE), the estimates of the direct effect (DE), and proportion of mediation (IE/DE + IE). The effects represent a relative change (%) in placental mtDNA content for an increment between the 10^th^-90^th^ percentile of FT_3_. All models were adjusted for maternal age, pre-pregnancy BMI, gestational age, newborn’s sex, smoking status, parity, maternal education, ethnicity, and cord plasma insulin level. ^*^ *p*-value < 0.05

We performed mediation analysis to estimate the proportion of the associations between cord blood thyroid hormones and placental mtDNA content that might be mediated by changes in mtDNA methylation if the underlying causal assumptions of the mediation analysis are valid. We selected both *MT-RNR1* and *D-loop* as potential mediators because methylation levels at both regions were significantly associated with FT_3_ and FT_4_ as well as with placental mtDNA content (Fig. 1). While adjusting for the aforementioned variables, we estimated that *MT-RNR1* methylation mediated 77% [indirect effect +14.61% (95% CI 2.64 to 27.98%, *p* = 0.01)] and *D-loop* methylation mediated 47% [indirect effect +8.60% (95% CI 1.23 to 16.50%, *p* = 0.02)] of the positive association between FT_3_ and placental mtDNA content (Fig. 2). Mediation models with FT_4_ gave similar results but the estimated effect proportions were smaller compared with those of FT_3_ (54 and 24%, respectively) (Additional file 1: Figure S2).

### Sensitivity analysis

Certain CpGs were more associated with fetal thyroid hormones compared to others, underscoring the importance of methylation levels at specific CpG (Additional file 2: Table S1). For example, FT_3_ was associated with methylation levels at all three CpG sites of *D-loop* (the transcription start site of the mitochondrial genome).

Excluding women who had undergone a cesarean section (*n* = 13) slightly increased our effect estimates for FT_3_ (Additional file 2: Table S2), whereas excluding small for gestational age infants (*n* = 22) slightly decreased our effect estimates for FT_3_ (Additional file 2: Table S3). However, we verified whether the association between fetal thyroid hormones and placental mtDNA content was still present in the initial group of 547 mother–child pairs (flowchart Additional file 1: Figure S3). Adjusting for the aforementioned variables, the association between cord blood FT_3_ and placental mtDNA content was stronger compared with the reported association in the main analysis, i.e., relative change of +29.22% (95% CI 16.22 to 43.67%, *p* < 0.0001) for a 10th–90th percentile increment in cord blood FT_3_, whereas the association for FT_4_ was less (+6.65%, 95% CI 2.23 to 11.26%, *p* = 0.003).

Furthermore, additional adjustments for cord blood plasma estradiol (*n* = 304), passive indoor tobacco smoke exposure (*n* = 295), alcohol consumption (*n* = 295), or pH of the arterial cord blood (indicator of hypoxemia) (*n* = 264), did not alter our associations between fetal thyroid hormones and mtDNA content or mtDNA methylation (data not shown).

Mitochondrial biogenesis relies on a tightly coordinated process between the nuclear and mitochondrial genome. For example, the nuclear gene *PPARGC1A* is a central regulator of mitochondrial gene expression and biogenesis that is also controlled by FT_3_ [14]. We observed an inverse association between fetal FT_3_ levels and placental *PPARGC1A* promoter methylation, i.e., −2.31% (95% CI −4.04 to −0.59, *p* = 0.009) for a 10th–90th percentile increment in FT_3_ (Additional file 2: Table S1). No association was observed between placental *PPARGC1A* promoter methylation and mtDNA content (*p* = 0.23) or mtDNA methylation (*D-loop*, *p* = 0.54; *MT-RNR1*, *p* = 0.74).

## Discussion

In this study, we report for the first time associations between fetal thyroid hormones and epigenetic modification at specific loci in the mitochondrial genome that could, at least in part, mediate the fetal thyroid-dependent regulation of mitochondrial biogenesis in placental tissue.

From mid-gestation onwards, the fetus starts secreting small amounts of thyroid hormones in addition to the transplacental supply of maternal thyroid hormones [15]. The optimal concentration of fetal thyroid hormones at each stage of development is maintained by the placenta, in conjunction with the fetal thyroid gland, liver, and kidneys [4]. The placenta expresses two types of iodothyronine deiodinases (D2 and D3) that are capable of metabolizing FT_3_ and FT_4_ [7], and thus, plays an important role in thyroid hormone homeostasis. Besides regulating fetal development [5], thyroid hormones have profound effects on mitochondrial energetics and biogenesis [1, 2].

Despite the spectacular progress in the knowledge of the T_4_-T_3_ nuclear pathway, a clear answer to how it regulates mitochondrial biogenesis is lacking. While it is known that an upregulation of nuclear-encoded respiratory genes including *PPARGC1A* occurs within hours after injection of T_3_ in hypothyroid rats, other non-genomic direct effects are detectable within minutes suggesting another mode of action [16, 17]. T_3_ binds directly to specific receptors inside the mitochondria. A 43 kDa c-Erb A alpha1 protein (p43), located exclusively in the mitochondrial matrix, acts as a T_3_-dependent transcription factor and specifically binds to four mitochondrial DNA sequences with a high similarity to nuclear T_3_ response elements [18, 19]. Overexpression of p43 increases mitochondrial genome transcription and protein synthesis, stimulating mitochondrial biogenesis in a T_3_-dependent manner [19]. Interestingly, of the four mitochondrial DNA sequences, two response elements are located in the *D-loop* region and one in the *MT-RNR1* gene. We hypothesized that DNA methylation at these mitochondrial hotspots could interfere with thyroid hormone-dependent regulation of mitochondrial biogenesis. First, we have shown that fetal thyroid hormones, especially FT_3_, are positively associated with placental mtDNA content and inversely associated with placental methylation levels at the *D-loop* and *MT-RNR1* region. Next, we underscored a substantial mediating role of placental mtDNA methylation between the association of fetal thyroid hormones and placental mtDNA content. Hence, we postulate that high DNA methylation levels in the mitochondrial genome are related to conformational or structural changes making the mtDNA less accessible to proteins and transcription factors such as the T_3_-dependent transcription factor p43. Previously, we have shown that exposure to particulate matter (PM_2.5_) during pregnancy is associated not only with changes in fetal thyroid hormones [20] but also with higher levels of methylation of the *D-loop* and the *MT-RNR1* sequence, affecting mtDNA content in placental tissue [13]. An increase in mtDNA methylation was observed in blood leukocytes of steel workers exposed to air pollution [21] and in an experimental study of human cultured cells treated with ethidium bromide [22]. The ethidium-bromide-exposed cells recovered from mtDNA depletion and showed increased overall methylation levels indicating that mtDNA was less packed with proteins during active mtDNA replication [22].

**Fig. 3:**
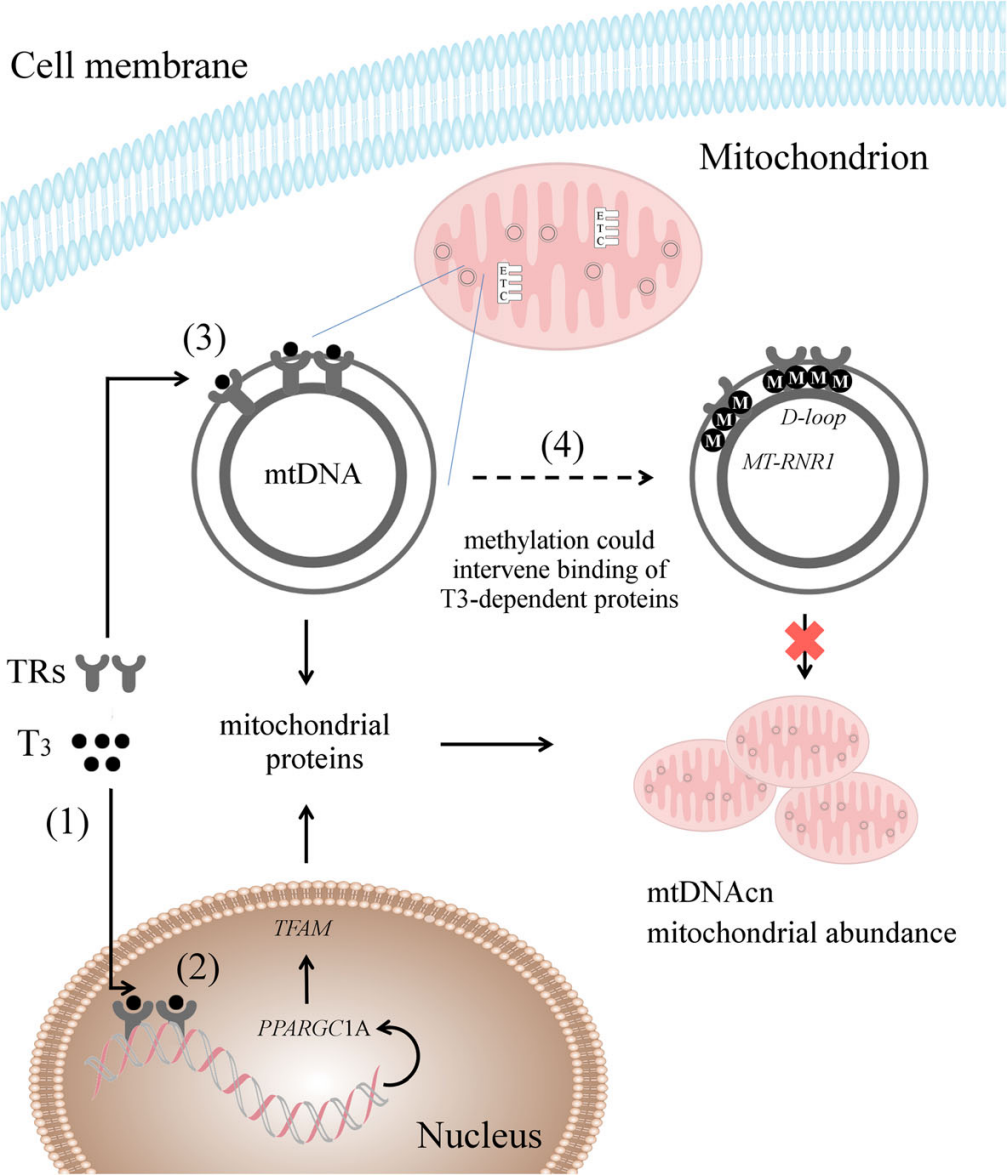
Simplified scheme of T_3_-dependent mitochondrial biogenesis through coordinated regulation of nuclear and mitochondrial gene products. T_3_ binds to thyroid receptors (TRs) (1) which consecutively bind to response elements in the nucleus activating expression of mitochondrial-related genes such as *PPARGC1A* (2). Alternatively, specific TRs are localized in the mitochondrial matrix (p43). The T_3_-p43 complex binds to response elements in the mitochondrial genome, of which two elements are located in the *D-loop* and one in the 12S rRNA (*MT-RNR1*) gene (3). We suggest that methylation of the mtDNA genome, in particular in the *D-loop* and *MT-RNR1* region, could intervene with T_3_-dependent mitochondrial protein production through conformational or structural changes making the mtDNA less accessible to proteins and transcription factors such as the T_3_-dependent transcription factor p43 (4)

Our mediation analysis revealed that the effect of FT_3_ on mtDNA content is mediated for 77% by *MT-RNR1* and for 54% by *D-loop* methylation levels, meaning that there are alternative routes, most likely nuclear pathways, by which thyroid hormones exert their action on mitochondrial biogenesis. T_3_ controls the expression of *PPARGC1A* [16], a transcriptional co-activator of several nuclear-encoded transcription factors including mitochondrial transcription factor A (*TFAM*) that regulates mitochondrial biogenesis [23] (Fig. 3). In addition to specific mitochondrial actions of FT_3_, we also observed an inverse association between placental *PPARGC1A* promoter methylation and fetal FT_3_ levels. Most likely this is indicative of upregulated *PPARGC1A* mRNA levels in the presence of high FT_3_ concentrations since promoter hypomethylation is usually associated with increased gene expression due to unwound DNA that is available for transcription factors. Interestingly, our interrogated region is a putative binding site for CREB transcription factors. The transcriptional activity of CREB is critical for the establishment and maintenance of energy homeostasis in mice neonates [24] and appears to be involved in the regulation of *PPARGC1A*. It is noteworthy to mention that the promoter region of *PPARGC1A* was largely unmethylated in cord blood but not in placental tissue [25], suggesting differential regulation of *PPARGC1A* in cord blood and placental tissue. Even though nuclear-encoded genes that are involved in mitochondrial function are regulated by epigenetic mechanisms [26], it would be interesting to determine the direct functional significance at mRNA expression level in response to FT_3_ levels. Unfortunately, we did not measure mRNA expression due to lack of suitable RNA samples and consider this a limitation of the study. We also have no clear information on “threshold” changes in mtDNA methylation that lead to functional effects of mitochondriogenesis. The mitochondrial genome, as far as we observed, shows a far less DNA methylation level (range 0 to 40%) compared with nuclear DNA (range 0 to 100%). Hence, small changes in mtDNA methylation might still have functional effects. It has been shown that small changes in single CpG sites and regional methylation changes interfere with gene/protein expression [27] by altering the affinity of transcription factors to their binding sites or chromosome looping events [28]. Even when small changes in methylation would not impact functional effects, mtDNA methylation can provide invaluable help in developing new and useful markers of exposure and disease [29]. However, our findings should first be confirmed in an experimental study and we need to investigate the health consequences in later life before we can translate it into a clinical meaning.

We are aware of the issue of placental tissue heterogeneity but it is difficult to assess the impact of this variation on our methylation results. To our knowledge, there is only one study that explored genome-wide DNA methylation patterns in the two main cell types of the placenta (cytotrophoblasts and fibroblasts) [30]. These cell types did not demonstrate genome-wide differentiation in DNA methylation but some specific genes showed differential promoter methylation. Although these authors demonstrated that the methylation profile of the placenta is mainly driven by cytotrophoblasts, there is a possibility that our epigenetic data is confounded by variation in cell type distributions. To overcome this obstacle, one should correct for differences in tissue composition using algorithms that estimate cell type proportions based on DNA methylation signatures derived from Illumina microarrays [31, 32]. However, statistical adjustments of DNA methylation data for cell distributions require the availability of reference epigenomes for the component cell types created by cell sorting [33]. Currently, there is no algorithm available to estimate the cell type proportions in placental tissue. Another limitation of our study is that we were not able to measure thyroid hormones in placental tissue but we used cord blood levels as a proxy since it circulates through the fetal side of the placenta.

## Conclusions

Given our epidemiological findings and other experimental research data, it seems that there exist coordinated events between mtDNA methylation, mtDNA content, and thyroid hormones. We summarized our findings in a simplified scheme (Fig. 3) indicating that epigenetic modification at specific loci of the mitochondrial genome could intervene with thyroid-dependent regulation of mitochondrial biogenesis. Our findings could contribute to further epidemiological understanding of mitochondrial disorders. Whether alterations in mitochondrial function or newborn’s thyroid hormone levels have health consequences later in life should be elucidated.

## Methods

### Study population

Within the on-going ENVIR*ON*AGE birth cohort (ENVIRonmental influence *ON* early AGEing) [34], we conducted our investigation in a group of 589 singleton pregnancies for which the mothers agreed withdrawal of cord blood, the collection of the placenta after delivery, and the use of information from their medical files. The placenta could not be collected for ten newborns, four placentas had insufficient DNA yield, 12 placentas had missing measurements of mtDNA content, and 16 mothers with thyroid gland complications were excluded. Because mtDNA methylation was measured only in a subset of this large study sample (*n* = 547), we ended with a final sample size of 305 newborns for the main analysis (see flowchart; Additional file 1: Figure S3).

Mother-child pairs were recruited from February 2010 to June 2014 at the East-Limburg Hospital in Genk (Belgium) following procedures approved by the Ethical Committee of Hasselt University and the East-Limburg Hospital and according to the principles outlined in the Helsinki Declaration for investigation of human subjects as previously described in details [35]. Briefly, written informed consent was obtained from eligible participants before delivery. Questionnaires and medical records consulted after birth provided information on maternal age, maternal education, smoking status, ethnicity, pre-pregnancy BMI, gestational age, newborn’s sex, Apgar scores, birth weight and length, parity, and ultrasonographic data. Information about maternal tobacco smoke exposure was obtained by asking whether mothers smoked during pregnancy, whether they smoked at any time during their life, or if they never smoked in their life. All neonates were assessed for congenital anomalies immediately after birth and were considered healthy with an Apgar score after 5 min ranging between 7 and 10. No neonate was delivered in the Neonatal Intensive Care Unit. The ENVIR*ON*AGE birth cohort generally consists of mothers with normal pregnancies without complications and with healthy neonates.

### Cord blood collection and thyroid hormones measurements

Immediately after delivery, the umbilical cord was clamped and cord blood was drawn in plastic BD Vacutainer® Lithium Heparin Tubes (BD, Franklin Lakes, NJ, USA). Blood tubes were centrifuged at 3200 rpm for 15 min to retrieve plasma which was instantly kept at −80 °C. FT_4_ (pmol/L), FT_3_ (pmol/L), TSH (mU/L), and insulin were measured in plasma using an electro-chemiluminescence immunoassay using the Modular E170 automatic analyzer (Roche, Basel, Switzerland) at the clinical lab of East-Limburg Hospital.

### Placental collection

Placentas were deep-frozen within 10 min of delivery, and afterwards, placental specimens were taken for DNA extraction after minimally thawing of the placentas. We took villous tissue (1 to 2 cm^3^) at a fixed location from the fetal side of the placenta, approximately 1–1.5 cm below the chorio-amniotic membrane, and preserved the biopsies at −80 °C [25]. Genomic DNA was isolated from placental tissue using the QIAamp DNA mini kit (Qiagen, Inc., Venlo, the Netherlands) and stored at −80 °C until further use.

### DNA methylation analyses

In 305 placentas, we performed DNA methylation analysis by highly quantitative bisulfite-PCR pyrosequencing as previously described [25]. Briefly, bisulfite conversions were performed using 1 μg of extracted genomic DNA with the EZ-96 DNA methylation Gold kit (Zymo Research, Orange, CA, USA) according to the manufacturer’s instructions. We interrogated CpG sites within specific regions of the mitochondrial genome (*MT-RNR1* and *D-loop*) and the promoter of *PPARGC1A* as described by Byun et al. [21] and Janssen et al. [25]. Detailed information regarding primer sequences is given in Additional file 2: Table S4. PCR amplification of regions of interest prior to pyrosequencing was performed in a total reaction volume of 30 μl, using 15 μl GoTaq Hot Start Green Master Mix (Promega, Madison, WI, USA), 10 pmol forward primer, 10 pmol reverse primer, 1 μl bisulfite-treated genomic DNA, and water. PCR products were purified and sequenced by pyrosequencing using the PyroMark Q96 MD Pyrosequencing System (Qiagen, Inc., Germantown, MD, USA). The degree of methylation was expressed as the percentage of methylated cytosines over the sum of methylated and unmethylated cytosines. Samples were run in duplicate on two different plates from which the average methylation levels were used. The coefficient of variation was 3.7% for *MT-RNR1*, 5.8% for *D-loop*, and 8.1% for *PPARGC1A*. The efficiency of the bisulfite-conversion process was assessed using non-CpG cytosine residues within the sequence. We used 0% (PSQ-T oligo: 5′-TTGCGATACAACGGGAACAAACGTTGAATTC-3′) and 100% (PSQ-C oligo: 5′-TTGCGATACGACGGGAACAAACGTTGAATTC-3′) DNA methylation control oligos. The sequencing primer for the control oligo was 5′-AACGTTTGTTCCCGT-3′. We mixed the PSQ-C oligo (or PSQ-T oligo) with the sequencing oligo in PyroMark Annealing Buffer (Qiagen, Inc., Valencia, CA, USA) and performed pyrosequencing with the sequencing entry C/TGTAT. The between- and within-placenta variability, exemplified by the intra-class correlation coefficient, was evaluated in a subset of 19 placentas and was 58 vs. 42% (*p* = 0.009) for *MT-RNR1*, 61 vs. 39% (*p* = 0.01) for the *D-loop* region and 64 vs. 36% (*p* = 0.005) for *PPARGC1A* [25].

### mtDNA content analysis

mtDNA content was measured in placental tissue by determining the ratio of two mitochondrial gene copy numbers (*MTF3212/R3319* and *MT-ND1*) to two single-copy nuclear control genes (*RPLP0* and *ACTB*) using a quantitative real-time polymerase chain reaction (qPCR) assay as previously described [35] but with small modification. Briefly, 2.5 μl diluted genomic DNA (5 ng/μl) was added to 7.5 μl mastermix consisting of Fast SYBR® Green I dye 2× (5 μl/reaction), forward and reverse primer (each 0.3 μl/reaction), and RNase free water (1.9 μl/reaction). Primer sequences (Additional file 2: Table S5) were diluted to a final concentration of 300 nM in the master mix. Samples were run in triplicate in 384-well format. Real-time PCR was performed using the 7900HT Fast Real-Time PCR System (Applied Biosystems, Foster City, CA, USA) with the following thermal cycling profile: 20 s at 95 °C (activation), followed by 40 cycles of 1 s at 95 °C (denaturation) and 20 s at 60 °C (annealing/extension), ending with melting curve analysis (15 s at 95 °C, 15 s at 60 °C, 15 s at 95 °C). qBase software (Biogazelle, Zwijnaarde, BE) was used to normalize data and correct for run-to-run differences [36].

### Statistical analysis

For database management and statistical analysis, we used the SAS software program (version 9.4; SAS Institute Inc., Cary, NC, USA). mtDNA content and thyroid hormone levels were log_10_-transformed to improve normality. For each subject, we measured in placental tissue methylation levels of CpG sites at two regions of the mitochondrial genome and at the promoter region of *PPARGC1A*. The pyrosequencing-based DNA methylation analysis produced a methylation value (%) for each CpG of *MT-RNR1* (two CpGs), the *D-loop* region (three CpGs), and *PPARGC1A* (three CpGs). In the main analysis, we used the average methylation levels of the different CpGs. Pearson correlation coefficients were calculated between the different thyroid hormone levels in cord blood (FT_3_, FT_4_, and TSH), mtDNA methylation (*MT-RNR1* and *D-loop*), and mtDNA content in placental tissue using R software packages. We performed multiple linear regression to determine the association between thyroid hormones and placental mtDNA methylation (both *MT-RNR1* and *D-loop*) and between thyroid hormones and placental mtDNA content. Thyroid hormones were fitted as linear variables in the models, and effect estimates on mtDNA methylation and mtDNA content were calculated for a 10th–90th percentile increment in thyroid hormones, which corresponds to a 52% change in FT_3_, a 12% change in FT_4_, and a 84% change in TSH. All models were adjusted for maternal age, pre-pregnancy BMI, gestational age, newborn’s sex, smoking status, parity, maternal education, ethnicity, and cord plasma insulin level. The Shapiro-Wilk statistic and Q-Q plots of the residuals were used to test the assumptions of all linear models.

We used mediation analysis to investigate potential associations that may underlie the relation between the exposure variable (FT_3_ or FT_4_) and the continuous outcome variable (mtDNA content) by examining how they relate to a third variable, the mediator (mtDNA methylation) [37]. We accomplished this by decomposing the total effect into a direct effect (DE; exposure effect on outcome at a fixed level of the mediator) and an indirect effect (IE; exposure effect on outcome that operates through the mediator).

### Sensitivity analysis

To underscore the importance of single CpG sites, we performed separate linear regression analyses between levels of FT_3_ or FT_4_ and the methylation levels at each CpG site of *MT-RNR1* and *D-loop*. Furthermore, it is known that cord blood thyroid hormone levels are influenced not only by different external factors such as fetal stress exemplified by cesarean section [38] but also by other factors such as cord plasma estradiol [39], passive smoking [40], alcohol consumption [41], and pH of arterial cord blood [42]. Besides adjusting the main model with the aforementioned variables, we performed additional analyses in which we excluded women who had undergone a cesarean section or excluding infants born with a birth weight less than the 10th percentile. Lastly, we used linear regression analyses to investigate the association between fetal thyroid hormones and placental promoter methylation of the nuclear gene *PPARGC1A*.

## Additional files

Additional file 1: Additional figures. **Figure S1.** Correlation plot between *MT-RNR1* and *D-loop* mtDNA methylation levels. The displayed methylation levels are absolute percentages. **Figure S2.** Estimated proportion of effects of FT_4_ exposure on mtDNA content mediated by mtDNA methylation. **Figure S3.** Flowchart depicting the selection for arriving at the final study sample either for placental mtDNA content measurements (*n* = 547) or for mtDNA methylation analysis (*n* = 305). (DOCX 520 kb)

Additional file 2: Additional tables. **Table S1.** Associations of cord blood thyroid hormones FT_3_ and FT_4_ with CpG-specific mtDNA methylation and *PPARGC1A* promoter methylation. **Table S2.** Associations of fetal thyroid hormones with placental mtDNA methylation and mtDNA content while excluding women who had undergone a cesarean section (*n* = 13). **Table S3.** Associations of fetal thyroid hormones with placental mtDNA methylation and mtDNA content while excluding small for gestational age infants (*n* = 22). **Table S4.** Bisulfite-pyrosequencing primer sequence information based upon Assembly GRCh37/hg19 of the UCSC genome browser. **Table S5.** Mitochondrial and nuclear primer sequence information based upon Assembly GRCh37/hg19 of the UCSC genome browser. (DOCX 54 kb)
