# Supplementary material for: Regulating role of fetal thyroid hormones on placental mitochondrial DNA methylation: epidemiological evidence from the ENVIRONAGE birth cohort study
Source: Clin Epigenetics. 2017 Jun 21;9:66. doi: 10.1186/s13148-017-0366-y (PMC5479026; doi:10.1186/s13148-017-0366-y)
Supplement: Supplementary file 1 — Additional figures. Figure S1. Correlation plot between MT-RNR1 and D-loop mtDNA methylation levels. The displayed methylation levels are absolute percentages. Figure S2. Estimated proportion of effects of FT4 exposure on mtDNA content mediated by mtDNA methylation. Figure S3. Flowchart depicting the selection for arriving at the final study sample either for placental mtDNA content measurements (n = 547) or for mtDNA methylation analysis (n = 305). (DOCX 520 kb) [file 13148_2017_366_MOESM1_ESM.docx]

Additional file 1: Additional figures





**Additional file 1: Figure S1.** Correlation plot between *MT-RNR1* and *D-loop* mtDNA methylation levels. The displayed methylation levels are absolute percentages.


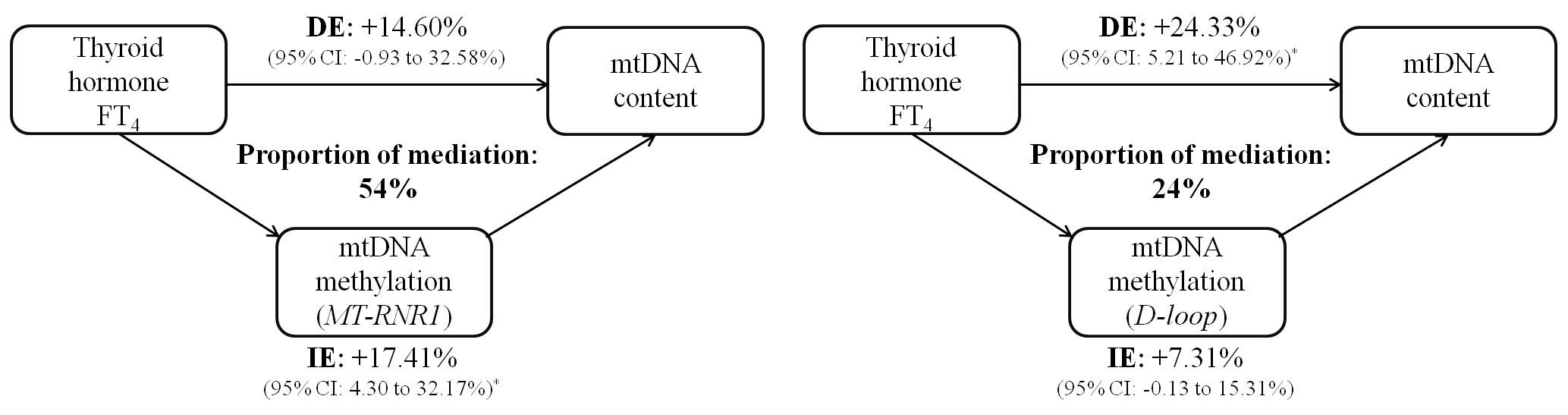


**Additional file 1: Figure S2.** Estimated proportion of effects of FT_4_ exposure on mtDNA content mediated by mtDNA methylation. The figure displays placental mtDNA methylation as mediator (left panel: *MT-RNR1*; right panel: *D-loop*), the estimates of indirect effect (IE), the estimates of the direct effect (DE), and proportion of mediation (IE/DE+IE). The effects represent a relative change (%) in placental mtDNA content for an increment between the 10^th^-90^th^ percentile of FT_4_. All models were adjusted for maternal age, pre-pregnancy BMI, gestational age, newborn’s sex, smoking status, parity, maternal education, ethnicity, and cord plasma insulin level. ^*^*p*-value < 0.05.


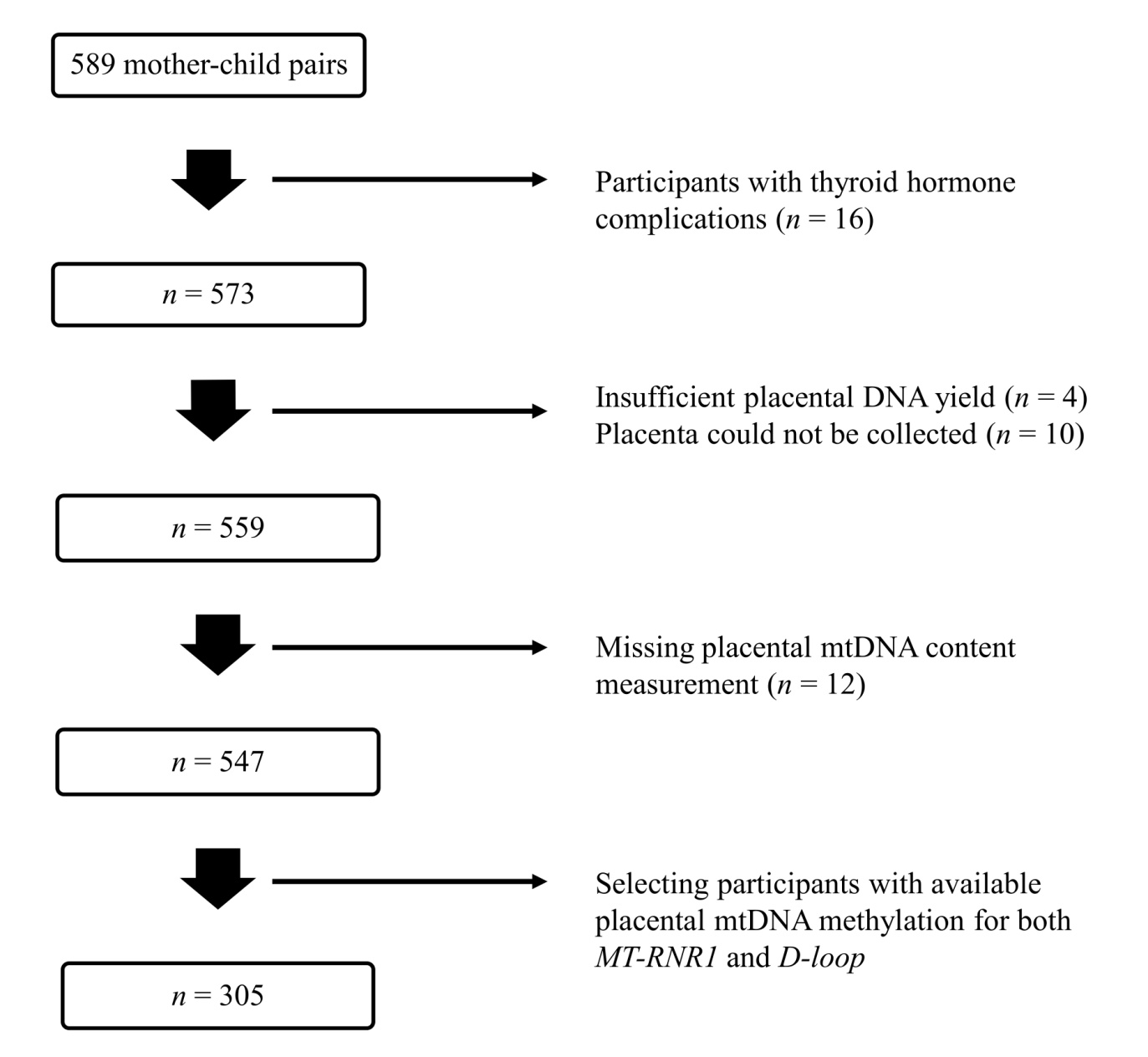


# **Additional file 1:** Figure S3. Flowchart depicting the selection for arriving at the final study sample either for placental mtDNA content measurements (*n* = 547) or for mtDNA methylation analysis (*n* = 305)
