# Supplementary material for: Regulating role of fetal thyroid hormones on placental mitochondrial DNA methylation: epidemiological evidence from the ENVIRONAGE birth cohort study
Source: Clin Epigenetics. 2017 Jun 21;9:66. doi: 10.1186/s13148-017-0366-y (PMC5479026; doi:10.1186/s13148-017-0366-y)
Supplement: Supplementary file 2 — Additional tables. Table S1. Associations of cord blood thyroid hormones FT3 and FT4 with CpG-specific mtDNA methylation and PPARGC1A promoter methylation. Table S2. Associations of fetal thyroid hormones with placental mtDNA methylation and mtDNA content while excluding women who had undergone a cesarean section (n = 13). Table S3. Associations of fetal thyroid hormones with placental mtDNA methylation and mtDNA content while excluding small for gestational age infants (n = 22). Table S4. Bisulfite-pyrosequencing primer sequence information based upon Assembly GRCh37/hg19 of the UCSC genome browser. Table S5. Mitochondrial and nuclear primer sequence information based upon Assembly GRCh37/hg19 of the UCSC genome browser. (DOCX 54 kb) [file 13148_2017_366_MOESM2_ESM.docx]

# Additional file 2: Additional tables

# Additional file 2: Table S1. Associations of cord blood thyroid hormones FT_3_ and FT_4_ with CpG-specific mtDNA methylation and *PPARGC1A* promoter methylation.

|  | **FT_3_, pmol/L** | |  | **FT_4_, pmol/L** | |
| --- | --- | --- | --- | --- | --- |
| **Gene** | **β^†^** | **(95% CI)** |  | **β^‡^** | **(95% CI)** |
| *MT-RNR1* |  |  |  |  |  |
| CpG1 | -0.46 | (-1.05 to 0.13) |  | -0.33 | (-0.54 to -0.11)^**^ |
| CpG2 | -2.54 | (-4.41 to -0.67)^*^ |  | -0.88 | (-1.56 to -0.19)^*^ |
| *D-loop* |  |  |  |  |  |
| CpG1 | -0.24 | (-0.46 to -0.02)^*^ |  | -0.07 | (-0.15 to 0.01) |
| CpG2 | -0.73 | (-1.32 to -0.14)^*^ |  | -0.23 | (-0.45 to -0.02)^*^ |
| CpG3 | -0.41 | (-0.74 to -0.08)^*^ |  | -0.11 | (-0.23 to 0.01) |
| *PPARGC1A* |  |  |  |  |  |
| CpG1 | -1.85 | (-3.29 to -0.41)^*^ |  | -0.02 | (-0.55 to 0.52) |
| CpG2 | -2.12 | (-3.96 to -0.29)^*^ |  | 0.08 | (-0.60 to 0.76) |
| CpG3 | -2.96 | (-5.46 to -0.46)^*^ |  | 0.21 | (-0.71 to 1.13) |
| Average CpGs^§^ | -2.31 | (-4.04 to -0.59)^*^ |  | 0.09 | (-0.55 to 0.73) |
| ^†^ β represents an absolute change in methylation (%) for a 10^th^-90^th^ percentile increment of FT_3_ cord blood thyroid hormone which corresponds to a 53% change in FT_3_.  ^‡^ β represents an absolute change in methylation (%) for a 10^th^-90^th^ percentile increment of FT_4_ cord blood thyroid hormone which corresponds to a 12% change in FT_4_.  ^§^ Average (10^th^-90^th^ percentile) methylation levels of *PPARGC1A* was 7.95% (2.77-16.45).  All models are adjusted for maternal age, pre-pregnancy BMI, gestational age, newborn’s sex, smoking status, parity, maternal education, ethnicity, and cord plasma insulin level.  ^*^ *p*-value < 0.05, ^**^ *p-*value *<* 0.005. | | | | | |

# Additional file 2: Table S2. Associations of fetal thyroid hormones with placental mtDNA methylation and mtDNA content while excluding women who underwent a cesarean section (*n* = 13).

|  | **FT_3_, pmol/L** | |  | **FT_4_, pmol/L** | |  | **TSH, mU/L** | |
| --- | --- | --- | --- | --- | --- | --- | --- | --- |
| **Variable** | **β** | **(95% CI)** |  | **β** | **(95% CI)** |  | **β** | **(95% CI)** |
| mtDNA methylation^†^ |  |  |  |  |  |  |  |  |
| *MT-RNR1*, % | -1.60 | (-2.84 to -0.35)^*^ |  | -0.52 | (-0.98 to -0.06)^*^ |  | 0.39 | (-0.21 to 0.99) |
| *D-loop*, % | -0.49 | (-0.88 to -0.11)^*^ |  | -0.12 | (-0.26 to -0.03)^*^ |  | 0.12 | (-0.07 to 0.30) |
| mtDNA content, %^‡^ | 24.37 | (3.09 to 50.06)^*^ |  | 12.64 | (5.09 to 20.35)^**^ |  | -5.16 | (-13.29 to 3.74) |
| ^†^ β represents an absolute change in percentage (%) methylation for a 10^th^-90^th^ percentile increment of thyroid hormone.  ^‡^ β represents a relative change (%) in placental mtDNA content for a 10^th^-90^th^ percentile increment of thyroid hormone.  All models are adjusted for maternal age, pre-pregnancy BMI, gestational age, newborn’s sex, smoking status, parity, maternal education, ethnicity, and cord plasma insulin level. ^*^ *p*-value < 0.05. | | | | | | | | |

#

# Additional file 2: Table S3. Associations of fetal thyroid hormones with placental mtDNA methylation and mtDNA content while excluding small for gestational age infants (*n* = 22).

|  | **FT_3_, pmol/L** | |  | **FT_4_, pmol/L** | |  | **TSH, mU/L** | |
| --- | --- | --- | --- | --- | --- | --- | --- | --- |
| **Variable** | **β** | **(95% CI)** |  | **β** | **(95% CI)** |  | **β** | **(95% CI)** |
| mtDNA methylation^†^ |  |  |  |  |  |  |  |  |
| *MT-RNR1*, % | -1.39 | (-2.65 to -0.13)^*^ |  | -0.64 | (-1.09 to -0.18)^*^ |  | 0.51 | (-0.10 to 1.13) |
| *D-loop*, % | -0.43 | (-0.81to -0.04)^*^ |  | -0.14 | (-0.28 to -0.0001)^*^ |  | 0.13 | (-0.06 to 0.32) |
| mtDNA content, %^‡^ | 16.54 | (-3.58 to 40.86) |  | 11.10 | (3.80 to 18.92)^**^ |  | -5.31 | (-13.67 to 3.87) |
| ^†^ β represents an absolute change in percentage (%) methylation for a 10^th^-90^th^ percentile increment of thyroid hormone.  ^‡^ β represents a relative change (%) in placental mtDNA content for a 10^th^-90^th^ percentile increment of thyroid hormone.  All models are adjusted for maternal age, pre-pregnancy BMI, gestational age, newborn’s sex, smoking status, parity, maternal education, ethnicity, and cord plasma insulin level. ^*^ *p*-value < 0.05. | | | | | | | | |

# Additional file 2: Table S4. Bisulfite-pyrosequencing primer sequence information based upon Assembly GRCh37/hg19 of the UCSC genome browser.

| **Gene symbol** | **Chr** | **Amplicon**  **start-end** | **Primer set (Forward / Reverse / Sequence)** | **Amplicon (bp)** | **Annealing temp (°C)** | **Target CpGs** |
| --- | --- | --- | --- | --- | --- | --- |
| *MT-RNR1* | M | 1191- | F: 5’-TTTTTAGAGGAGTTTGTTTTGTAAT-3’ | 176 | 58.3 | 2 |
|  | (+) | 1366 | R: 5’-ATAACCCATTTCTTACCACCTCATA-3’ |  |  |  |
|  |  |  | S: 5’-AGTTTGTTTTGTAAT-3’ |  |  |  |
| *D-loop* | M | 6- | F: 5’-TGTGTAGATATTTAATTGTTATTA-3’ | 254 | 54.1 | 3 |
|  | (-) | 259 | R: 5’-CAAATCTATCACCCTATTAACCAC-3’ |  |  |  |
|  |  |  | S: 5’-TAATTAATTAATATATTT-3’ |  |  |  |
| *PPARGC1A* | 4 | 23891766- | F: 5’-TTTTTTGTGTAGTTTGTTGTTTTAA-3’ | 231 | 53 | 3 |
|  |  | 23891997 | R: 5’TTACAAAAAATTTTAATTATTATATAACCA-3’ |  |  |  |
|  |  |  | S: 5’-AGTTTGTTGTTTTAATT-3’ |  |  |  |

Abbreviations: *MT-RNR1*: 12S ribosomal RNA; *D-loop*: Displacement loop; *PPARGC1A*: peroxisome proliferator-activated receptor γ-coactivator1α.

# Additional file 2: Table S5. Mitochondrial and nuclear primer sequence information based upon Assembly GRCh37/hg19 of the UCSC genome browser.

| **Gene symbol** | **Chr** | **Amplicon**  **start-end** | **Primer set (Forward / Reverse)** | **Amplicon (bp)** | **Primer efficiency**  **(%)** |
| --- | --- | --- | --- | --- | --- |
| *MTF3212/R3319* | M | 3213- | F:5’-CACCCAAGAACAGGGTTTGT-3’ | 108 | 96.3 |
|  |  | 3320 | R:5’-TTAACAACATACCCATGGCCA-3’ |  |  |
| *ND1* | M | 3314- | F:5’-ATGGCCAACCTCCTACTCCT-3’ | 115 | 99.3 |
|  |  | 3428 | R:5’-AAAGGCCCCAACGTTGTAG-3’ |  |  |
| *RPLP0* | 12 | 120636904- | F:5’-CCCAATTGTCCCCTTACCT-3’ | 85 | 100.7 |
|  |  | 120636988 | R:5’-GAACACAAAGCCCACATTCC-3’ |  |  |
| *ACTB* | 7 | 5567833- | F:5’-ACTCTTCCAGCCTTCCTTCC-3’ | 102 | 96.8 |
|  |  | 5567934 | R:5’-TGTGGAAGCTAAGTCCTGCC-3’ |  |  |
| Abbreviations: *MTF3212/R3319:* Mitochondrial forward primer from nucleotide 3212 and reverse primer from nucleotide 3319; *ND1:* Mitochondrial encoded NADH dehydrogenase 1; *RPLP0:* Acidic ribosomal phosphoprotein P0; *ACTB:*Beta actin*.* | | | | | |
